# Research Progress on the Reaction of Carbon Dioxide with Hydrazones and Their Derivatives

**DOI:** 10.3390/molecules30091987

**Published:** 2025-04-29

**Authors:** Hong-Xia Sun, Shao-Xuan Gong, Hong-Yang Zhang, Yu-Ting Liu, Li-Ling Shi, Yong-Jie Zhu, Xiu-Mei Xie, Jun-Jie Li, Jing Wen, Yong-Chang Guan, Zhen Zhang, Miao Zhang, Yun-Feng Zhang

**Affiliations:** 1 Natural Products Chem-Bio Innovation Center, College of Food and Biological Engineering, Chengdu University, Chengdu 610106, China; 13320964726@163.com (H.-X.S.); gongshaoxuan@stu.cdu.edu.cn (S.-X.G.); lyuting398@gmail.com (Y.-T.L.); shililing0201@foxmail.com (L.-L.S.); yongjiezhu76@gmail.com (Y.-J.Z.); 13281296726@163.com (X.-M.X.); 18685820575@163.com (J.-J.L.); wenjing02154784@163.com (J.W.); 2 Department of Applied Biology and Chemical Technology, Research Institute for Smart Energy, The Hong Kong Polytechnic University, Hung Hom, Hong Kong 999077, China; hongyang.zhang@polyu.edu.hk (H.-Y.Z.); guanyc0317@sina.com (Y.-C.G.); 3 School of Chemistry and Chemical Engineering, Shanxi University, Taiyuan 030006, China; 4 The Hong Kong Polytechnic University Shenzhen Research Institute, Shenzhen 518057, China

**Keywords:** carbon dioxide (CO_2_), hydrazones, carbamates, umpolung strategy, cyclization, lactamization

## Abstract

CO_2_, an abundant and renewable C1 source, presents significant potential for applications in organic synthesis. Hydrazones, recognized for their distinctive properties, exhibit high versatility in synthetic chemistry, facilitating numerous chemical transformations. Given their crucial roles in organic synthesis, the combination of CO_2_ with hydrazones has garnered increasing research interest. This review provides a comprehensive summary of recent progress in reactions involving CO_2_ and hydrazones or their derivatives. These include the coupling of amines and N-tosylhydrazones with CO_2_, the umpolung-mediated carboxylation of hydrazones/N-tosylhydrazones with CO_2_, the cyclization of hydrazones with CO_2_, and lactamization reactions incorporating N-tosylhydrazones and CO_2_. These transformations utilize the diverse reactivity of hydrazones and their derivatives to capture and convert CO_2_, generating valuable organic compounds with both academic and practical relevance. Additionally, the review examines the mechanisms underlying these reactions, offering critical insights for advancing research in this area.

## 1. Introduction

Carbon dioxide (CO_2_), a major greenhouse gas associated with global warming, has attracted widespread attention for its capture and utilization. As an abundant and renewable C1 feedstock, CO_2_ offers significant potential for applications in organic synthesis [1,2,3,4,5]. Its low cost, wide availability, and environmentally sustainable nature make it an appealing option for green chemistry. However, the activation and efficient utilization of CO_2_ in organic reactions present substantial challenges due to its intrinsic thermodynamic stability and kinetic inertness, which impede its involvement in conventional chemical processes. Despite these obstacles, significant advancements have been made in developing strategies for CO_2_ activation and conversion [6,7,8,9,10,11,12,13,14,15,16,17,18,19,20,21,22,23,24,25,26].

Hydrazones are formed through the condensation of aldehydes or ketones with hydrazine or its derivatives, possessing the general structure RR′C=N-NHR″. Their distinctive properties are highly useful in synthetic chemistry, facilitating participation in diverse chemical transformations. For example, they act as intermediates in the Wolff–Kishner reduction and play a crucial role in the synthesis of Barton vinyl iodides, among other synthetically valuable reactions [27]. Recent studies by Li and other researchers have led to significant progress, highlighting the importance of hydrazone-based compounds in organic synthesis [28,29,30,31]. Among various hydrazone derivatives, N-tosylhydrazones have gained prominence due to their role as operationally safe carbene precursors. Considerable advancements have been reported in transition metal-catalyzed reactions, metal-free conditions, and photocatalytic processes under light irradiation [32,33,34,35]. Furthermore, hydrazones have been extensively utilized in cyclization reactions, facilitating the synthesis of heterocyclic compounds containing N-N moieties, which hold significant value in organic chemistry [36].

**Figure 1:**
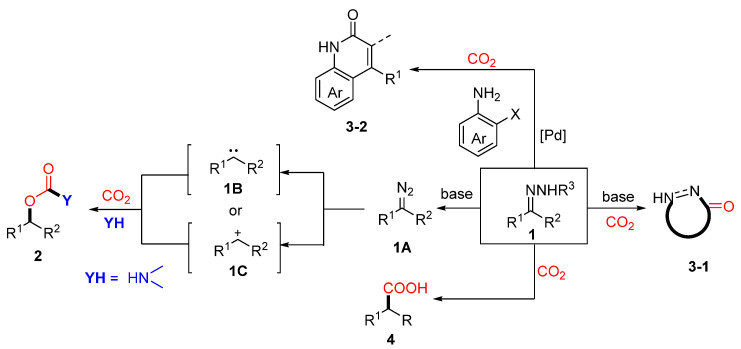
Overview of CO_2_-involved transformations of hydrazones and their derivatives.

Given the essential roles of hydrazones (**1**) in coupling, cyclization, and polarity-reversal reactions, along with the significance and challenges associated with CO_2_ conversion, research efforts have increasingly focused on integrating hydrazone chemistry with CO_2_ utilization and fixation. By exploiting the distinctive reactivity of hydrazones, a variety of CO_2_-involved transformations have been developed, facilitating the synthesis of valuable compounds such as carbamates (**2**), carbonyl compounds (**3-1**, **3-2**), and organic carboxylic acids (**4**) (Figure 1).

This review highlights recent progress in reactions involving CO_2_ and hydrazones or their derivatives, with an emphasis on how the diverse chemical properties of hydrazones contribute to different modes of CO_2_ fixation. Additionally, the scope and mechanisms of these reactions are analyzed to provide a deeper understanding of their underlying principles. Through this comprehensive examination, valuable perspectives and potential directions for the future advancement of CO_2_ utilization are proposed.

## 2. Reaction of Carbon Dioxide with Hydrazones and Their Derivatives

### 2.1. Coupling of Amines and N-tosylhydrazones with CO_2_ to Generate Carbamates

**Figure 2:**
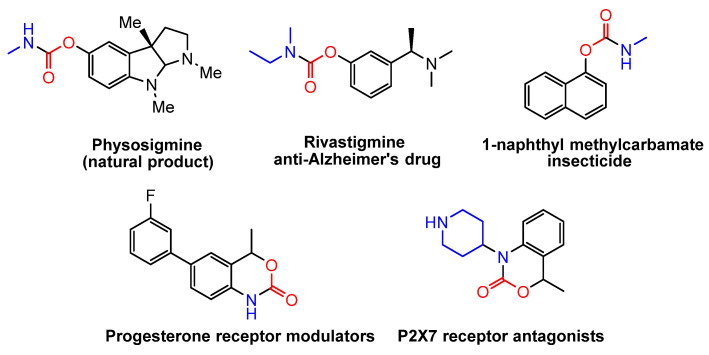
Representative pharmaceuticals containing the carbamate motif.

Organic carbamates are an important class of compounds with significant biological and pharmaceutical properties, frequently found in natural products [37,38,39,40,41,42,43,44,45,46,47] (Figure 2). Their synthesis has been extensively investigated. Conventionally, carbon monoxide (CO), phosgene, triphosgene, and isocyanates have been commonly utilized as carbonyl sources for their construction [40,41,42,43,44]. Additionally, CO_2_ has been explored as a starting material for carbamate synthesis [45,46,47,48,49,50,51,52,53]. This approach primarily involves the nucleophilic attack of amines on CO_2_, leading to the formation of carbamic acids (or their salts), which subsequently undergo coupling to afford the target products.

N-tosylhydrazones are widely recognized as synthetic intermediates in the formation of carbon–carbon or carbon–heteroatom bonds through both transition metal-catalyzed and metal-free cross-coupling reactions [37,54,55]. Under protonic conditions, diazo compounds generated in situ from N-tosylhydrazones undergo decomposition, yielding carbocation intermediates [56].

**Figure 3:**
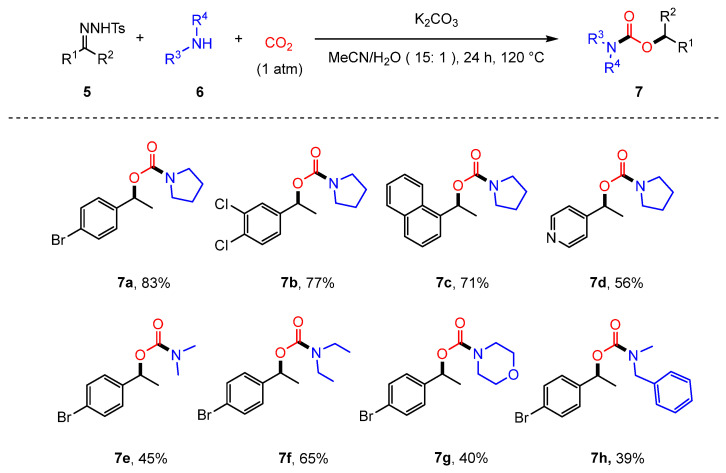
Base-promoted coupling of CO_2_, amines, and N-tosylhydrazones for carbamate synthesis.

**Figure 4:**
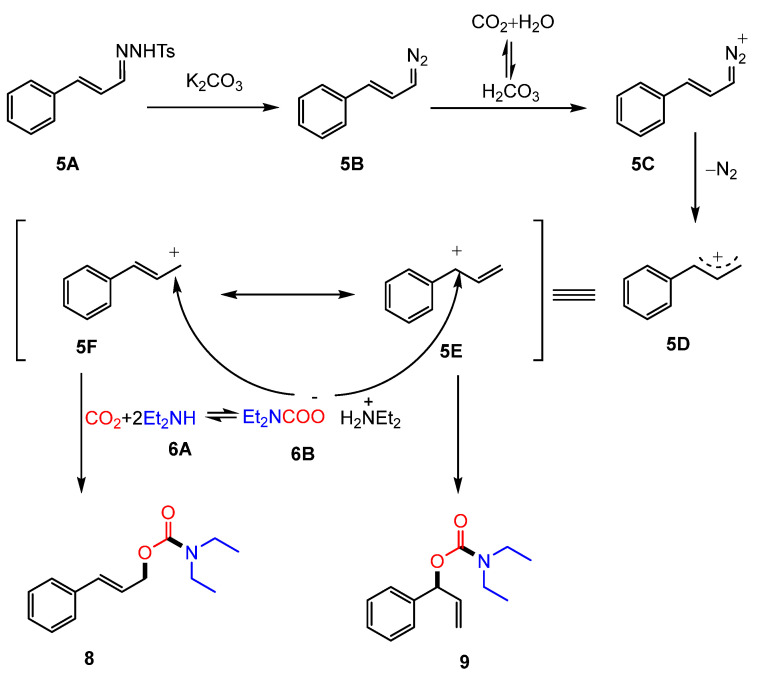
Mechanism of carbamate formation through a carbocation intermediate.

In 2015, JIANG and co-workers reported a base-promoted coupling reaction involving CO_2_, amines (**6**), and N-tosylhydrazones (**5**) [57]. Under basic conditions, amines—particularly secondary amines with strong nucleophilicity—interact with CO_2_ to generate carbamate salts. These salts are then captured by carbocation intermediates generated in situ from N-tosylhydrazones, enabling an efficient synthesis of diverse carbamate esters (**7**). This reaction system also accommodates primary amines, although the yields are comparatively lower (Figure 3). However, N-tosylhydrazones derived from aliphatic ketones or aldehydes and aromatic amines did not yield the desired products. Mechanistic investigations and control experiments confirmed the presence of a carbocation intermediate (**5D**) and demonstrated that both H_2_O and CO_2_ facilitate the protonation process through forming carbonic acid, promoting carbocation intermediate formation from the diazo compound (Figure 4).

**Figure 5:**
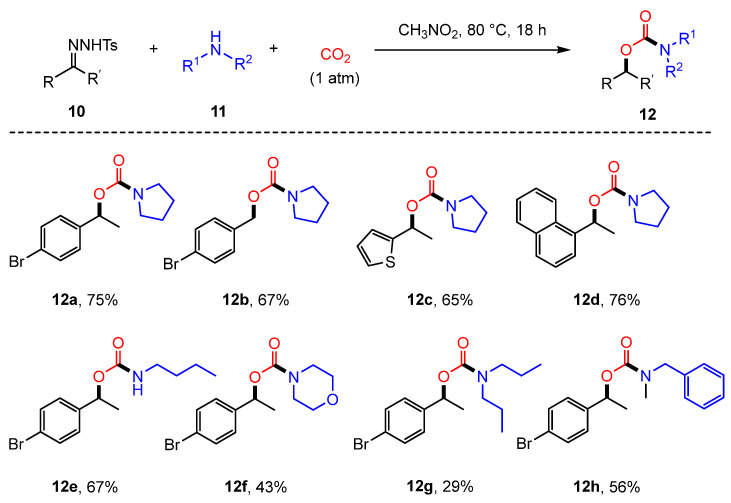
Synthesis of carbamates under 1 atm CO_2_ without an external base.

Owing to the intrinsic basicity of amine compounds, they can function as bases, enabling the formation of carbamate salts without requiring additional bases. In 2016, Chung et al. successfully carried out this reaction under 1 atm of CO_2_ without an external base, employing nitromethane as the solvent [58]. Notably, the reaction conditions allowed for scalability, enabling synthesis on a gram scale. This study also included several examples of reactions involving primary amines, with most yields observed within the moderate range (Figure 5).

The authors propose that the reaction proceeds via a mechanism in which the amine (**11**) reacts with CO_2_ in situ to generate a carbamate species (**12**), which acts as a nucleophile to couple with the N-tosylhydrazone (**10**), ultimately yielding the carbamate product. This mechanistic pathway eliminates the need for high-pressure conditions and external bases, offering a milder and more efficient synthetic approach compared to conventional methods (Figure 5).

**Figure 6:**
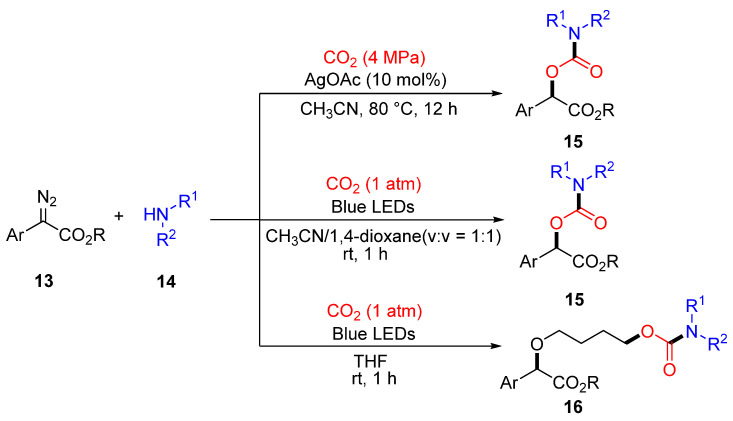
The reaction of diazonium and CO_2_ under different conditions.

Here, it is worth noting that diazo compounds are recognized as pivotal intermediates in the aforementioned reaction and have attracted considerable attention due to their versatile reactivity in diverse chemical transformations. The Jiang and Qi successfully developed a silver-catalyzed or photocatalytic three-component coupling reaction involving α-diazoesters (**13**), CO_2_, and amines (**14**), which enabled the efficient synthesis of α-carbamoyloxy esters (**15, 16**) [59,60]. Notably, under photocatalytic conditions using tetrahydrofuran (THF) as the reaction medium, an exclusive four-component coupling reaction was observed between α-aryldiazoesters, amines, CO_2_, and THF. This process resulted in the formation of a wide range of structurally diverse carbamate products (Figure 6).

The above reactions, due to the use of different catalytic systems, exhibit slightly different reaction path. Under AgOAc catalysis, α-diazo esters decompose to generate silver carbene intermediates, which then undergo multi-step coupling with CO_2_ and amines to form α-carbamates. The mechanism involves carbene insertion into CO_2_ and nucleophilic attack by amines, followed by protonation to afford the product [59]. In contrast to the silver-catalyzed system, blue light excitation of α-diazo esters generates carbene intermediates, with the solvent (THF or 1,4-dioxane/MeCN) dictating the reaction pathway: in THF, the carbene forms an oxonium ylide with the solvent, which then combines with the carbamate anion generated from amines and CO_2_; in the mixed solvent, the carbene directly couples with the carbamate anion without metalcatalysis [60].

**Figure 7:**
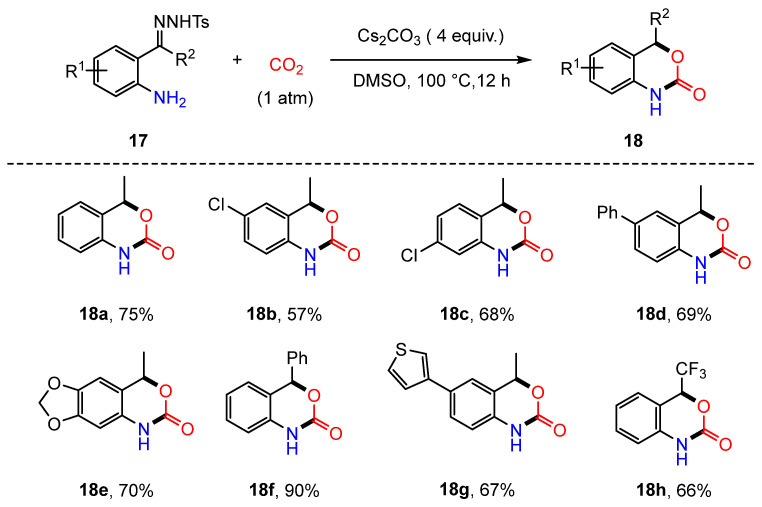
Intramolecular cyclization of *o*-aminoacetophenone N-tosylhydrazone with CO_2_.

**Figure 8:**
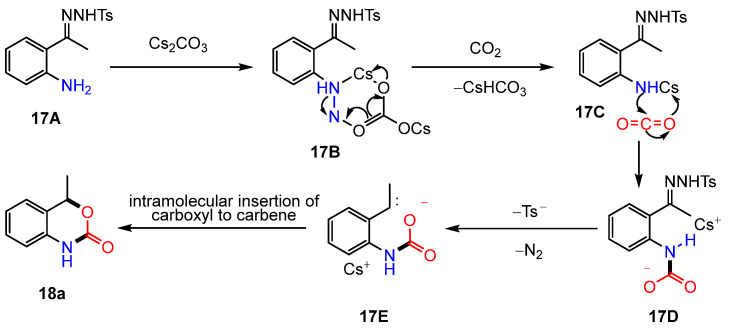
Mechanism of intramolecular cyclization with CO_2_.

The previous discussion primarily addressed intermolecular reactions; however, intramolecular processes had not been reported until 2019, when Cheng et al. introduced a novel strategy for incorporating CO_2_ into *o*-aminoacetophenone N-tosylhydrazone derivatives (**17**). This approach enabled the synthesis of a series of 1,4-dihydro-2H-3,1-benzoxazin-2-one compounds (**18**) using Cs_2_CO_3_ [61] (Figure 7). The proposed reaction mechanism is illustrated in Figure 8. Initially, Cs_2_CO_3_ interacts with aniline, promoting proton abstraction and generating intermediate **17C**. This is followed by the carboxylation of the amine with CO_2_, leading to the formation of intermediate **17D**. Subsequently, intermediate **17D** undergoes a stepwise elimination of Ts and N_2_, facilitated by the base, resulting in the formation of a carbene intermediate (**17E**). Finally, the carboxyl group undergoes an intramolecular insertion into the carbene intermediate, yielding the 1,4-dihydro-2H-3,1-benzoxazin-2-one products (Figure 8).

In summary, this method presents several advantages, including the use of readily available starting materials, broad substrate scope, mild reaction conditions, and operational simplicity. It offers an efficient and practical approach for synthesizing a diverse range of organic alkyl carbamate esters.

### 2.2. Carboxylation of Hydrazones/N-Tosylhydrazones with CO_2_ Through Umpolung

**Figure 9:**
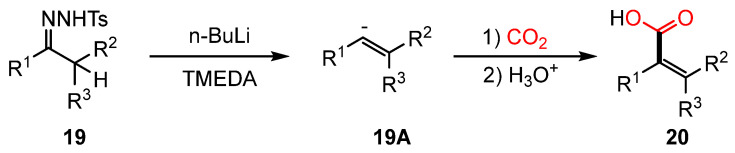
Traditional Shapiro reaction for α-arylacrylic acid synthesis.

The Umpolung strategy modifies the inherent electronic characteristics of functional groups, effectively reversing their typical polarity. This polarity shift enables the emergence of novel reactivity patterns, allowing the formation of new chemical bonds [62,63,64,65]. A well-known example is the Shapiro reaction, in which an N-tosylhydrazone (**19**) undergoes deprotonation by a base, generating a vinyllithium intermediate. This intermediate subsequently reacts with electrophiles, leading to the formation of alkene-based products (**20**). When CO_2_ is employed as the electrophile, α-arylacrylic acids can be synthesized [66,67,68,69,70,71,72,73]. Conventionally, these reactions require the use of a strong base (e.g., *n*-BuLi) and extremely low temperatures, which has restricted their practical applicability (Figure 9).

**Figure 10:**
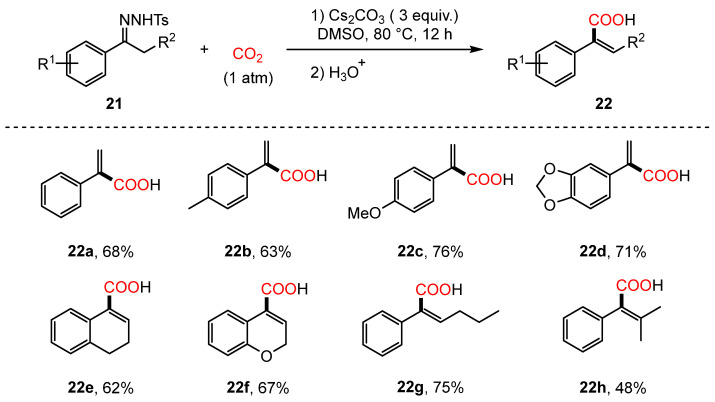
Cs_2_CO_3_-promoted carboxylation of N-tosylhydrazones using CO_2_.

In 2015, Cheng and colleagues developed a Cs_2_CO_3_-mediated carboxylation reaction between N-tosylhydrazones (**21**) and CO_2_, providing an efficient approach for synthesizing α-arylacrylic acids (**22**) [74]. This method serves as a practical and elegant alternative to the conventional Shapiro reaction. Through systematic optimization and control experiments, it was demonstrated that CO_2_ functions as the carbonyl source in this transformation. The reaction exhibits compatibility with a range of aromatic ring substitution patterns; however, substrates bearing strong electron-withdrawing groups did not afford the desired products, indicating possible interference with the reaction mechanism (Figure 10).

**Figure 11:**
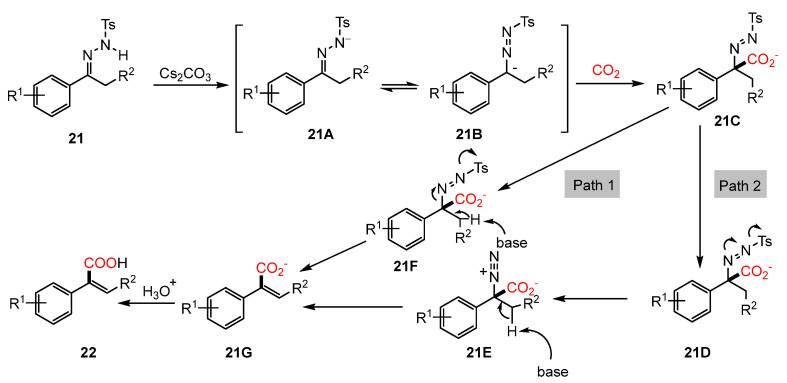
Mechanism of Cs_2_CO_3_-promoted carboxylation of N-tosylhydrazones.

The reaction mechanism involves the in situ formation of a diazo intermediate from N-tosylhydrazones, catalyzed by Cs_2_CO_3_ under mild conditions. Initially, Cs_2_CO_3_ promotes deprotonation of the hydrazone substrate, generating a nitrogen-centered anion, which subsequently undergoes isomerization to form a carbanion. This carbanion then captures CO_2_, forming an intermediate that undergoes desulfonylation, leading to the generation of a diazo intermediate. Under basic conditions, the diazo species undergoes nitrogen extrusion, followed by protonation, ultimately yielding the α-arylacrylic acid product (Figure 11).

The key parts of the mechanism of this reaction are as follows: (i) deprotonation by Cs_2_CO₃ generating a nitrogen-centered anion; (ii) isomerization to carbanion enabling CO_2_ capture; and (iii) desulfonylation and N_2_ extrusion yielding α-arylacrylic acids.

**Figure 12:**
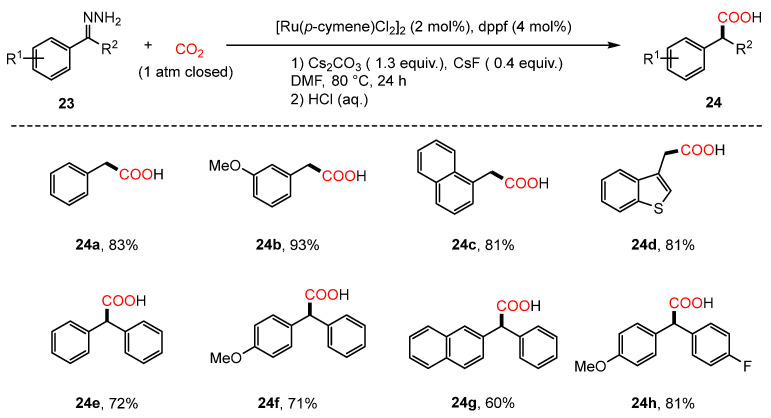
Ru-catalyzed umpolung carboxylation of hydrazones involving CO_2_.

**Figure 13:**
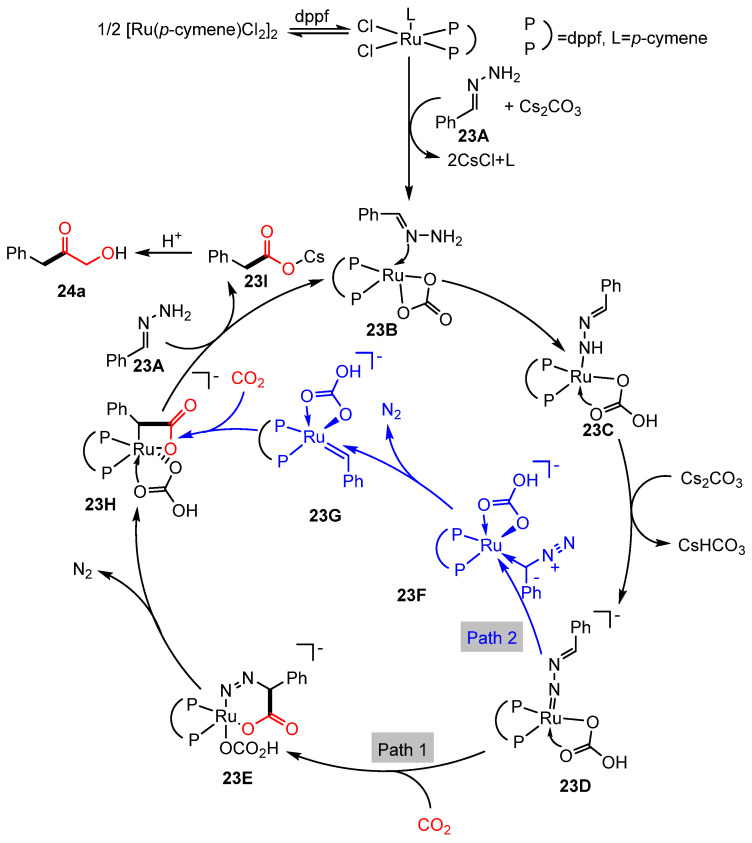
Mechanism of Ru-catalyzed umpolung carboxylation reaction.

The described process operates as a base-promoted reaction. Expanding upon Li’s work on Ru-catalyzed umpolung reactions that utilize carbonyls as carbanion equivalents [28], Yu, Lan, and Li introduced a novel strategy in 2018 for synthesizing aryl acetic acids (**24**). This approach involves the cleavage of C=N double bonds in hydrazones through Ru-catalyzed umpolung reactions [75] (Figure 12). In these transformations, the air-stable ligand dppf (1,1’-Bis(diphenylphosphino)ferrocene) was identified as optimal for efficient aryl acetic acid synthesis. Furthermore, reaction conditions were refined to accommodate less reactive hydrazones derived from ketones. A proposed mechanism, supported by experimental findings and Density Functional Theory (DFT) calculations, suggests that the reaction initiates with ligand exchange between the ruthenium catalyst and phenylhydrazone in the presence of Cs_2_CO_3_, forming a ruthenium–hydrazone complex(**23B**). This complex subsequently undergoes a sequence of protonation steps, generating a Ru-nitrenoid intermediate(**23D**). A [4 + 2] cycloaddition between this intermediate and CO_2_ then forms a six-membered Ru complex(**23E**), which, upon nitrogen extrusion and protonation, regenerates the catalyst and releases the target aryl acetic acid. Additionally, an alternative mechanistic pathway has been proposed, wherein isomerization, carbene formation, and CO_2_ insertion result in the formation of an alternative intermediate complex (Figure 13).

**Figure 14:**
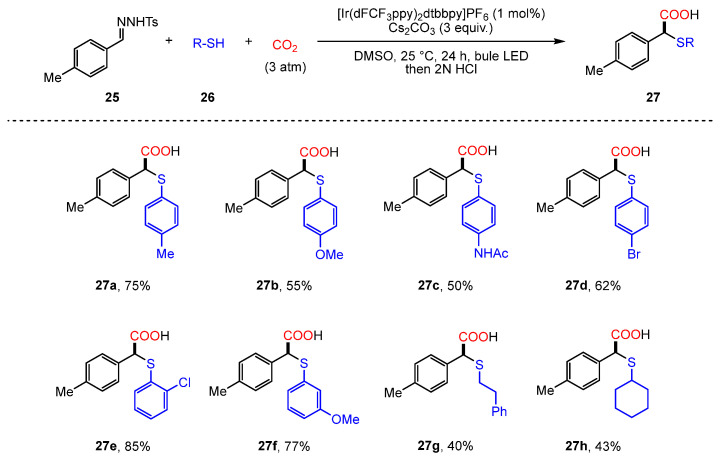
Photoredox-catalyzed difunctionalization of tosylhydrazones with CO_2_.

In 2020, König and colleagues introduced a significant advancement by integrating photoredox catalysis with the Wolff–Kishner reaction to achieve the difunctionalization of N-tosylhydrazones (**27**) using CO_2_ [76] (Figure 14). This reaction follows a three-component mechanism involving preformed N-tosylhydrazones, thiols, and CO_2_.

**Figure 15:**
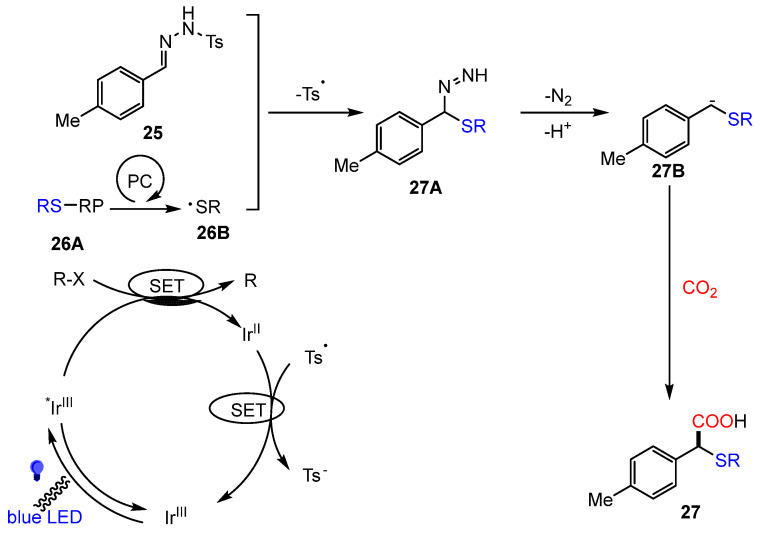
Mechanism of photoredox-catalyzed difunctionalization reaction.

The proposed mechanism involves: (i) Photoredox-generated thiyl-radical addition to N-tosylhydrazone; (ii) Base-assisted formation of diazene intermediate; (iii) Carbanion trapping by CO_2_ to afford α-thioether carboxylic acids. A key aspect of the mechanism is the photoredox-mediated generation of a thiyl radical (**26B**), which subsequently adds to the N-tosylhydrazone substrate (**25**). This step is followed by the formation of a diazene intermediate (**27A**), which undergoes base-promoted nitrogen extrusion to generate a carbanion (**27B**). CO_2_ then functions as the electrophile, capturing the carbanion and completing the difunctionalization process (Figure 15). Through this method, a variety of α-thioether-functionalized carboxylic acids were synthesized under relatively mild conditions, highlighting the potential for practical applications.

### 2.3. Cyclization of Hydrazones with CO_2_

**Figure 16:**
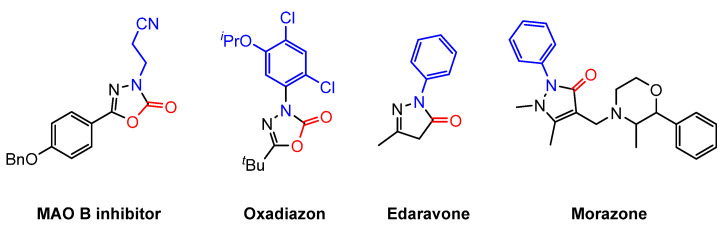
Representative structures containing the carbonyl-containing azole motif.

The significance of nitrogen-containing heterocyclic compounds is well recognized. Carbonylation reactions utilizing carbon dioxide offer a greener and safer alternative to highly toxic reagents such as carbon monoxide and phosgene, contributing to the synthesis of carbonyl-containing heterocyclic compounds [11]. The nucleophilic nitrogen atom in hydrazones and their derivatives enables reactions with carbon dioxide, facilitating the formation of carbonyl-containing azole compounds (Figure 16).

**Figure 17:**
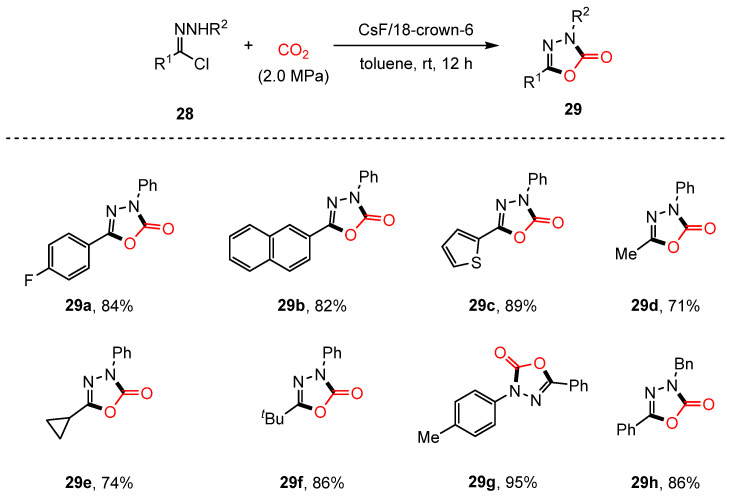
1,3-Dipolar cycloaddition of nitrilimines with CO_2_.

In 2017, Lv and colleagues developed an efficient methodology for synthesizing 1,3,4-oxadiazol-2(3H)-ones (**29**) through the 1,3-dipolar cycloaddition of nitrilimines (**28**) with carbon dioxide, catalyzed by CsF/18-crown-6 [77] (Figure 17). This strategy demonstrates broad substrate compatibility, enabling hydrazinyl chlorides with various substituents to participate in the reaction. The successful synthesis of a reversible MAO-B inhibitor and the commercial herbicide Oxadiazon further underscores the practical applicability of this approach.

Control experiments and NMR analysis revealed that 18-crown-6 plays a critical role in facilitating the formation of the nitrilimine intermediate, while the CsF/18-crown-6 system significantly enhances the reactivity of CO_2_.

**Figure 18:**
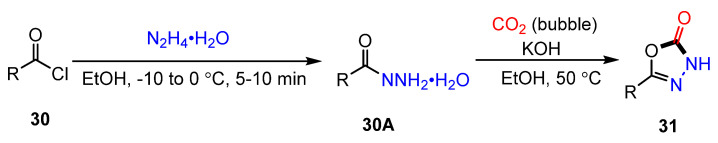
CO_2_-involved transformations involving diazo compounds.

Interestingly, these structures can also be synthesized via the carbonylation of hydrazides using CO_2_ as a reactant. A notable example is the work by Suen and colleagues in 2015, who demonstrated a KOH-mediated carbonylation reaction of hydrazides (**30A**) with CO_2_ [78]. The hydrazide precursors were readily obtained through the reaction of acid chlorides (**30**) with hydrazine monohydrate, offering a highly efficient synthetic route (Figure 18).

**Figure 19:**
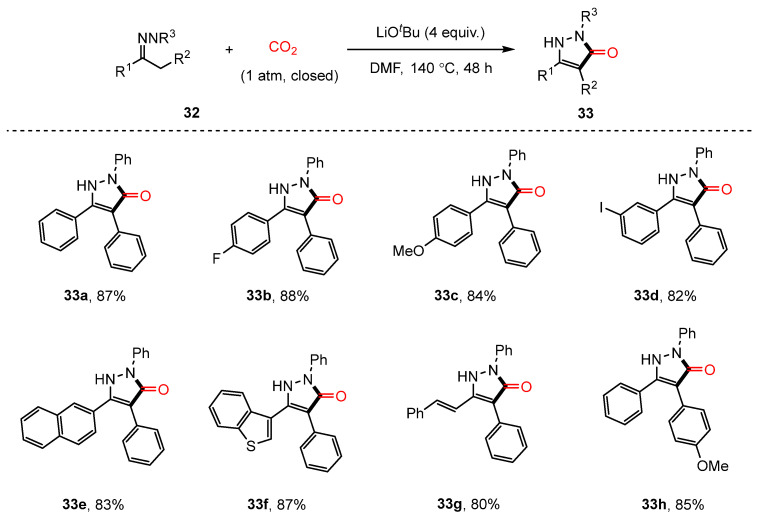
C(sp^3^)-H carbonylative cyclization reaction of hydrazones using CO_2_.

**Figure 20:**
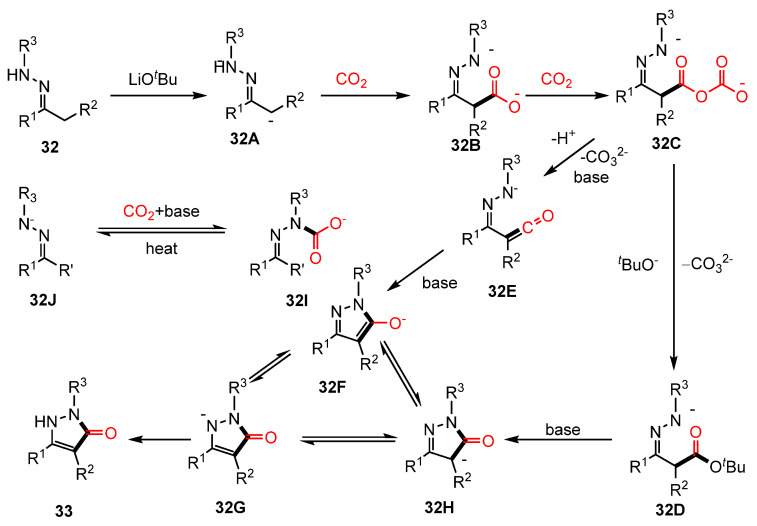
Mechanism of C(sp^3^)-H carbonylative cyclization reaction.

The carbonylation of C(sp^3^)–H bonds using carbon dioxide has attracted considerable interest; however, due to its inherent challenges, only a limited number of studies have been reported in this area [11]. Recently, Hu’s team successfully developed a method for the cyclization of hydrazones with CO_2_, enabling the synthesis of various pyrazolone derivatives via 1°, 2°, or 3° C(sp^3^)-H carbonylative cyclization reactions [79] (Figure 19). To evaluate the practical applicability of this reaction, synthesis was achieved on a gram scale, and several functional transformations were performed on the resulting pyrazolone derivatives (**33**). Notably, the successful synthesis of a PKC inhibitor with anti-cancer activity demonstrated the potential biomedical relevance of this approach. Experimental findings and previous literature suggested that mixed acid anhydride compounds **32C** serve as crucial intermediates. Under basic conditions, these intermediates can undergo transformations leading to the formation of either **32D** or **32E**, ultimately yielding the target cyclic products (Figure 20).

Overall, carbonylation reactions utilizing carbon dioxide have become a crucial approach for synthesizing carbonyl-containing heterocyclic compounds. The interaction of carbon dioxide with hydrazones and their derivatives frequently results in the formation of valuable carbonyl-containing azole compounds, which hold significant promise for broad applications in pharmaceuticals and materials science.

### 2.4. Lactamization Reaction of N-Tosylhydrazones, 2-Iodoanilines, and CO_2_

**Figure 21:**
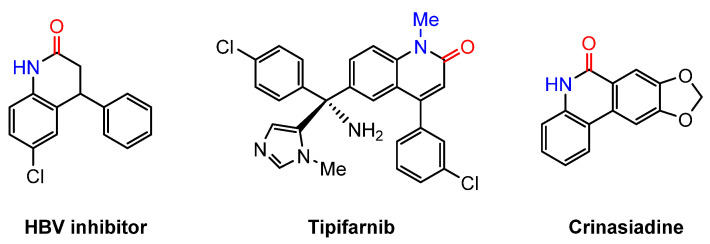
Representative structures containing the quinolinone motif.

Quinolinones are an important class of organic compounds with broad applications in pharmaceuticals and materials science (Figure 21).

**Figure 22:**
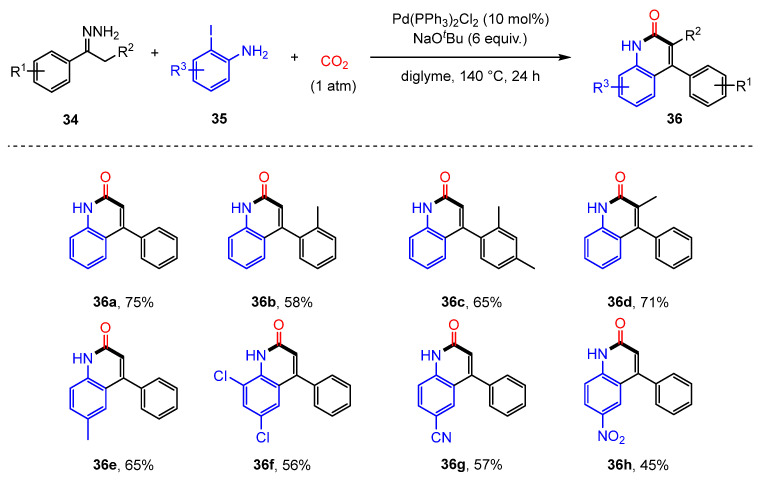
Pd-catalyzed synthesis route for 4-aryl-2-quinolinones.

In 2016, Yu’s group developed a method for synthesizing quinolinone derivatives via the lactamization of C(sp^2^)–H bonds with CO_2_ [80]. However, this approach requires the use of pre-synthesized *o*-alkenyl- or *o*-(hetero)arylanilines as substrates. Later that year, Cheng’s group introduced a palladium-catalyzed three-component reaction involving N-tosylhydrazones (**34**), 2-iodoanilines (**35**), and atmospheric CO_2_, enabling the efficient synthesis of a variety of 4-aryl-2-quinolinones (**36**) (Figure 22) [81]. This methodology allows the formation of two C-C bonds, one C=C bond, and one C-N bond within a single reaction vessel, providing a highly effective strategy for incorporating CO_2_ into heterocyclic frameworks.

**Figure 23:**
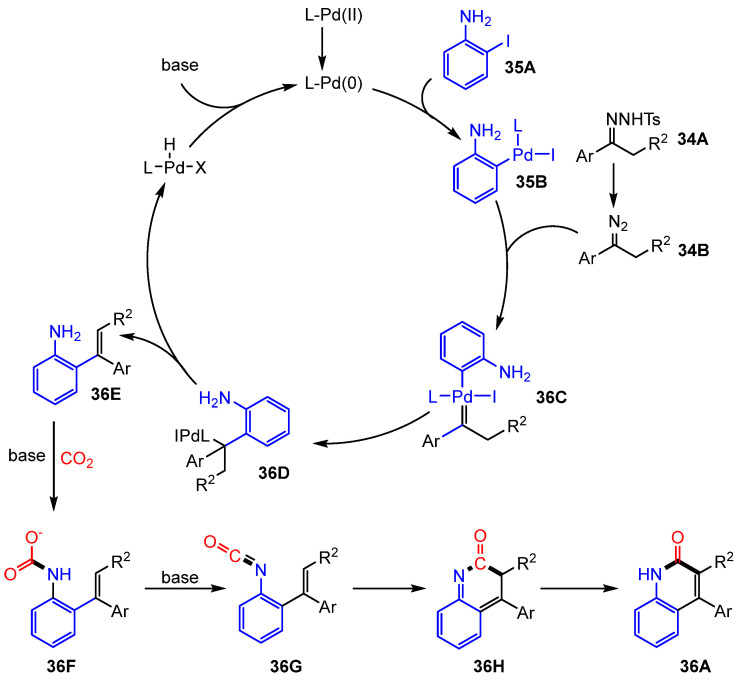
Mechanism of palladium-catalyzed three-component coupling reaction.

The proposed reaction mechanism begins with the palladium-catalyzed coupling of aryl halides and N-tosylhydrazones, leading to the formation of an *o*-vinyl aniline intermediate(**36E**). This intermediate subsequently undergoes C(sp^2^)-H lactamization with CO_2_, yielding the target quinolinone. Additionally, an alternative reaction pathway involving the formation of *o*-iodoisocyanatobenzene before the generation of *o*-vinyl aniline remains a possibility (Figure 23).

## 3. Conclusions

As research on carbon dioxide fixation and utilization continues to gain attention, the conversion of CO_2_ into high-value-added chemicals has emerged as a key focus. Hydrazone compounds and their derivatives, which serve as essential raw materials or intermediates in organic synthesis, represent a promising avenue for CO_2_ utilization when integrated with CO_2_ conversion strategies. Based on the reactions discussed in this paper, the primary products currently obtained include carbamates, organic carboxylic acid derivatives, and certain nitrogen-containing heterocycles. These compounds are widely utilized in pharmaceuticals, materials science, and as intermediates in organic synthesis, underscoring the relevance of combining CO_2_ utilization with hydrazone chemistry. The major reaction types involved include coupling, polarity inversion, and cyclization.

However, the development of this integration has not yet matched the progress in CO_2_ chemistry or hydrazone chemistry, and the range of reactions and products remains relatively limited. For instance, in Section 2.1, the predominant reaction described involves the coupling of nitrogen as a nucleophile with CO_2_ and phenylhydrazine to form carbamate compounds. Reactions involving other nucleophiles such as oxygen, sulfur, and carbon, which could potentially yield asymmetric carbonates or carboxylates, have not been explored, despite their significance in related fields. In Section 2.2, among the reaction types discussed, only ruthenium-catalyzed polarity inversion has been reported for the synthesis of organic carboxylic acids. The use of alternative metal catalysts, such as nickel and palladium, remains unexplored, with the primary products being aryl acetic acids and acrylic acids. Expanding the use of more cost-effective metals in this field could significantly enhance the applicability of these reactions. Furthermore, this section highlights photocatalytic transformations that successfully facilitate the formation of thioacids. The application of rational design strategies to achieve carboxylation reactions involving other heteroatoms, such as nitrogen, could further expand potential applications. Based on the current state of development, there remains substantial room for growth in the integration of CO_2_ chemistry with hydrazone chemistry.

Additionally, with the rapid advancements in photocatalysis and electrocatalysis as sustainable chemical methods, numerous transformations previously unattainable through traditional organic or transition metal catalysis have been realized, significantly advancing organic chemistry. The feasibility of applying photocatalysis and electrocatalysis to the CO2-involved conversion of hydrazone compounds presents an important avenue for further investigation.

## Data Availability

Not applicable.
